# Empowering Women in Organic Chemistry (EWOC) at Five Years: Giving Back and Getting Back

**DOI:** 10.1021/acscentsci.3c01136

**Published:** 2023-09-29

**Authors:** Elinor H. Cantor, Margaret M. Faul, Donna M. Huryn, Lara Kallander, Rebecca T. Ruck, Mary P. Watson

The Cambridge Dictionary defines *empowerment* as “the process of gaining freedom and power to do what you want or to control what happens to you”.^1^ In recent years, two publications have highlighted the scarcity of women in the field of medicinal chemistry^2^ and in process chemistry departments,^3^ leading areas of employment for trained organic chemists. At pharmaceutical companies and in other industries, both disciplines clock in at ∼20% representation. While this analysis does not account for all roles in academia, for example, organic chemistry professors,^4^ or other relevant areas of the workforce, it stands to reason that women are an underrepresented group in organic chemistry and would benefit from *empowerment*.^5^ In late 2017, the Empowering Women in Organic Chemistry (EWOC)^6^ movement was born to do just that and more, laying out the following ambitious goals:

- Establish a peer group network for collaborating and recruiting
- Afford a novel mechanism to provide advice and counsel for women organic chemists
- Provide guidance to graduate students and postdoctoral associates making career decisions
- Provide community support to enhance retention of women in chemistry

As EWOC Founders, when we embarked on this journey we saw the gap, the need, and the opportunity to establish this network and set about establishing an annual conference as a starting point for our efforts. What has emerged has not only achieved the aforementioned stated goals but also far exceeded our wildest expectations. As EWOC just celebrated its fifth conference, it seemed appropriate to look back at where we started and where we are headed, the impacts sought and achieved, the learnings gleaned and applied, and the opportunities to continue to advance our goals and elevate our contributions to the broader organic chemistry community.

## I How It Started

### I.A The Who

In the fall of 2017, Dr. Lara Kallander (then at GlaxoSmithKline) reached out to Prof. Donna Huryn (then at the University of Pittsburgh) after reading her article^2^ that encouraged “the creation of events ... specifically dedicated to women to allow mutual support” to discuss the possibility of creating an organic chemistry conference for women. Inspired by belonging research^7^ and the Grace Hopper Celebration,^8^ Kallander hypothesized that building community would improve the retention of women in chemistry. After discussion, Kallander and Huryn began to plan an event that would be an opportunity to attract, retain, and develop women through their careers in the field of organic chemistry. They leveraged their networks to identify additional individuals who would be excited and committed to the stated vision. Dr. Ellie Cantor joined the founding members, having connected to them through the Philadelphia chapter of the Association for Women in Science. Huryn had a relationship with Dr. Margaret Faul (Amgen) through years of being among the only women at conferences and, as author of the aforementioned treatise on women in medicinal chemistry,^2^ had advised Faul and Dr. Rebecca Ruck (Merck & Co., Inc., Rahway, NJ) on their manuscript on women in process chemistry.^3^ Both were quick to pledge their allegiance to EWOC. Finally, with an eye toward adding another academic to the equation, Ruck reached out to Prof. Mary Watson (University of Delaware), her former labmate during their postdoc and Ph.D., respectively, to complete the team. This assembly of the EWOC founders is a testament tothe importance of networking and networks, a key point of emphasis for EWOC, and to the corresponding connections formed by the committee over their careers.

### I.B The What

With this powerhouse team, a mission, and a vision, the next step was to translate these abstract concepts into something tangible and deliverable, specifically an answer to the question of what a conference would need to look like to be successful. The founders initially envisioned a weekend-long retreat comprised of talks, posters, networking, and workshops. While these activities aligned with our goals, we quickly identified two flaws with our logistical proposal: (1) scheduling on a weekend is less than compatible with personal commitments, a fact that is applicable to any conference but certainly in the case of a conference that aims to empower women; (2) for our first foray, a multiday event seemed overly ambitious, especially because of the additional travel costs involved for those brave enough to attend our first EWOC. With these considerations in mind, we settled on a single-day (Friday) first EWOC symposium that would combine the desired elements, with the optimistic perspective that success could foster modifications in both duration and programming.

### I.C Year One: June 28, 2019, at the University of Pennsylvania Department of Chemistry^9^

Given Prof. Huryn’s association with the University of Pennsylvania, where Dr. Kallander was also then serving as a Lecturer, the decision was taken to hold the inaugural conference at the Penn Department of Chemistry. This approach, combined with the generosity of Penn Chemistry, would serve to manage the budget by minimizing venue costs while positioning for a local group of attendees from nearby founder affiliations. The first line-up of presenters—Profs. Madeleine Joullié, Malika Jeffries-EL, Geraldine Richmond, Catherine Grimes, Dr. Emma Parmee, and Lisa Jarvis (Table 1)—was invited due to their demonstrated scientific excellence and experience as leading women in chemistry and represented a range of career options (academia, industry, and scientific writing), career stages, and areas of organic chemistry (synthesis, polymers, and chemical biology). They were asked to feature chemistry accomplishments and weave in elements of challenges endured and addressed in their careers. Five workshops were created to run across lunch, with the critical goal of providing information and building skill sets that would be beneficial toward personal career development and for positioning organizations to be better advocates for the targeted audience. Topics were (1) cultivating a sense of belonging, (2) networking, (3) creating confidence, (4) choosing a career path, and (5) driving cultural change.^10^ Fund-raising efforts allowed us to establish minimal registration fees and offset a considerable percentage of travel expenses for trainees attending the conference.

**Table 1:** Speakers across Five Years of EWOC

year	speaker	affiliation	discipline
2019	Prof. Catherine Grimes	University of Delaware	chemical biology
2019	Lisa Jarvis	C&EN	science communication
2019	Prof. Malika Jeffries-EL	Boston University	organic materials
2019	Prof. Madeleine Joullié	University of Pennsylvania,	synthetic chemistry
2019	Dr. Emma Parmee	Merck & Co., Rahway, NJ	medicinal chemistry
2019	Prof. Geraldine Richmond	University of Oregon	physical chemistry/COACh development
2020	Prof. Carolyn Bertozzi	Stanford University	chemical biology
2020	Prof. Donna Blackmond	Scripps	physical organic chemistry
2020	Prof. Vy Dong	University of California, Irvine	synthetic chemistry
2020	Prof. Julia Kalow	Northwestern University	organic materials
2020	Dr. Nicola Webb	Corteva Agriscience	process chemistry
2021	Dr. Margaret Chu-Moyer	Amgen	medicinal chemistry
2021	Prof. Shanina Sanders Johnson	Spelman College	synthetic chemistry
2021	Prof. Anne McNeil	University of Michigan	organic materials
2021	Prof. Sarah Reisman	Caltech	synthetic chemistry
2021	Prof. Alanna Schepartz	University of California, Berkeley	chemical biology
2022	Charlotte Allerton, MPhil	Pfizer	medicinal chemistry
2022	Prof. Abigail Doyle	UCLA	synthetic chemistry
2022	Prof. Davita Watkins	The Ohio State University	organic materials
2022	Prof. Alison Wendlandt	Massachusetts Institute of Technology	synthetic chemistry
2022	Prof. Christina Woo	Harvard University	chemical biology
2023	Dr. Margaret Faul	Amgen	process chemistry
2023	Prof. Laura Kiessling	Massachusetts Institute of Technology	chemical biology
2023	Prof. Jessica Kisunzu	Colorado College	synthetic chemistry
2023	Dr. Jennifer Petter	Arrakis Therapeutics	medicinal chemistry
2023	Prof. Melanie Sanford	University of Michigan	synthetic chemistry
2023	Prof. Seunghyun Sim	University of California, Irvine	metabolic engineering

With these logistical considerations in place, we identified several measures for success: attendance, a postconference survey, and our own anecdotal experience. With respect to conference size, we initially envisioned that 60 attendees would be sufficient to achieve our intent and position EWOC to grow in future years; we quickly exceeded that number of registrants and repeatedly increased the cap we had artificially installed. In a similar vein, we outgrew the intended lecture hall and quickly relocated to the largest chemistry auditorium at Penn. In the end, the 2019 EWOC conference hosted 170 attendees (at 94–95% she/her pronouns provided), far exceeding our wildest expectations for the brand-new event. While local (northeast and mid-Atlantic U.S.) representation comprised the bolus of participants, attendees traveled from across the country, including from Texas, New Mexico, and California.

This landscape served to cement the value of our mission, which was further reinforced in the conference survey. Of the 73 respondents, where a score of 5 equaled extremely likely to attend again and 4 was somewhat likely, the average score was 4.67. Attendees articulated their unprecedented experience at EWOC: the esteemed women speakers as scientific role models and leaders willing to make themselves vulnerable in this setting, the uniquely collaborative environment and positive energy cultivated by having so many women in organic chemistry together in one room, and the connections forged and confidence instilled via the framework. Several comments encapsulated this message:

- I loved the talks! They were perfect. I loved how they showed the evolution of careers from a variety of fields within chemistry, from how they became interested in science to the science they are working on today. It was inspiring. I’ve already told friends they need to attend next year!
- I’ve sat in a room with 20–30 other female medicinal chemists before, and I thought I would feel similarly in a room of ∼200 of them, but I was completely wrong. I was surprised that, by simply sitting in a large auditorium full of women in organic chemistry, I felt a sense of confidence and belonging that I had, frankly, never felt before. Right after I had that feeling, I had the realization that this is probably how men feel most of the time. It was pretty illuminating—thank you for bringing all of us together!
- I was uncertain whether I should attend this year or wait until the conference really took off. However, I’m so glad I attended this year because it exceeded my expectations—even just sitting in a room full of talented women with a passion for organic chemistry was extremely empowering. You have got a good thing going, and I cannot wait for future conferences!!

Additionally, a tweet from Jake Yeston of *Science* serves as a good reminder that EWOC is not only an opportunity to bring women together but also an opportunity for men to learn and become strong allies: “I like to think I’m unbiased. But really, every guy should have the experience of sitting in a room with just ten or so other guys and over a hundred women. They should understand viscerally what 10% or 20% representation means, how self-conscious it makes you.”^11^ This collective feedback mirrored the founders’ personal assessments, where we collectively felt a sense of validation and a sentiment of helping to change the future trajectory for women in chemistry.

We did receive three significant pieces of constructive feedback that we have sought to incorporate in subsequent iterations of EWOC. First, some felt that the talks lacked sufficient scientific content. As founders, we agreed with this sentiment, as we sought to emphasize the chemical wizardry of our presenters; ultimately, this has been addressed year-on-year. Second, having tried to pack a healthy combination of talks, workshops, a poster session, and ample networking in only 7.5 h, we realized the proceedings ended up feeling rushed, and attendees desired more time to interact with each other. Finally, not everyone felt the proposed sense of inclusion at EWOC, specifying a desire for more women of color presenters and men attendees, meaning it was necessary for us to be more intentional in subsequent years to invite a diversity of attendees to the conference table.

Following the success of the first EWOC conference, the founders approached the American Chemical Society’s Division of Organic Chemistry (DOC) about forging a mutually beneficial partnership for future iterations. The leaders from both sides agreed to work together to promote the event and install the infrastructure to ensure sustainability for EWOC. Another manifestation of this success has been an increase in the number of sponsors and levels of sponsorship from across the field. These financial contributions have been crucial to keeping attendee costs down and rendering EWOC as broadly accessible as possible, a theme that will be covered in greater depth in the next section.

## II How It’s Going

### II.A 2020,^12^ Year Two: In Person at Genentech → Virtual; 2021,^13^ Year Three: Virtual

For the second EWOC conference, we planned to increase the number of attendees to ∼250. To achieve that level of participation, the event was planned for Friday, August 14, 2020, at Genentech in South San Francisco; this siting and timing were intentional as the Fall ACS meeting was slated to begin Sunday, August 16, in San Francisco. Ultimately, this approach fell into a “best laid plans” scenario, whereby owing to the COVID-19 pandemic, the organizers made the difficult decision in June to pivot the conference to a virtual format. While effectively designing a new conference for a second year in a row felt like a herculean task, we deemed it critical to ensure that EWOC2020 happened to best capitalize on the success of our first year.

Given the transition to a virtual format, it was critical to assess how to maximize engagement in the core activities of EWOC, paying special attention to how to optimize virtual networking opportunities. The talks were the easiest piece here, with speakers and audience by then being very familiar with the Zoom interface (though we will always have to remind someone they are on mute!). The Q&A feature likewise allowed for straightforward audience engagement with the speakers, both immediately following the talks and with any additional questions in the queue. The same was true of the career panel, which ran in the middle of the day, while workshops buttressed the beginning and end of the day to accommodate disparate time zones, leveraging breakout sessions to enable small group interactions as needed. The poster session was moved to the evening before, with no limitation placed on the number of presenters. Finally, two unique networking sessions were chartered. After the Thursday poster session, a variety of themed virtual networking sessions ranging from career to diversity topics^14^ were held, allowing attendees to meet and connect with individuals with similar interests and potential mentors. There was also a closing networking session/happy hour at which attendees were randomly placed in a series of breakout rooms to meet additional new people and discuss all aspects of the conference. Uncertain how these virtual networking components would compare with the in-person version given the general inexperience with virtual conferences at the time, we were pleased to receive extremely positive feedback on the format:

- The virtual sessions were a great place to really get a feel for the community and relationships that have been formed between so many people, and it was great to see and be a part of that even if it was virtually.
- I really enjoyed the networking sessions that were hosted. I think that the virtual format was convenient because random (SMALL) groups could be easily generated.
- As a woman in chemistry, finding and connecting with others in the field is one of the most validating and supporting things you can do. It is an immensely useful tool for promoting retention of women in organic chemistry.
- I loved the feeling of support I got from this conference. There were so many people being very affirming of my own experiences, and I loved hearing about what others are interested in/struggling with. This felt like it was possible because there were workshops and networking sessions that were smaller in size, allowing for more personal connections.

All told, the format worked exceptionally well and demanded only small tweaks for our second virtual year with #EWOC2021, where the biggest change was the addition of “Meet Our Sponsors” sessions on the first day of the conference. These engagements arose from the increased number of sponsors in the EWOC rolodex and their corresponding desire to have an increased level of engagement with participants. With a certain level of financial contribution, sponsors were provided a Zoom room for a 15–30 min “virtual sales booth” with attendees. These sessions proved to be popular with students and postdocs who found them “very helpful, both in networking and making industry careers approachable and accessible”, and they are now a staple of Day 1 of EWOC.

In assembling the agendas for 2020 and 2021, we sought to address some of the feedback received from the respective prior year. The presenters selected were all better positioned to provide primarily scientific talks, with appropriate titration of their personal stories. Among these speakers, we paid greater attention to representation, including more women of color. The same was true for the career panel, where we likewise featured more women of color, along with women using their chemistry degrees across a range of sectors. Prof. Carolyn Bertozzi from Stanford University spoke of her experiences as a member of the LGBTQ+ community and how that has helped shape her career. Networking sessions focused on communities, such as Black, Indigenous & People of Color (BIPOC) Peer Networking, Allies and Advocates Peer Networking, and LGBTQ+ Peer Networking. Despite these efforts, there is plenty of room for continued improvement, and we hope that EWOC can help to cultivate this critical pipeline of talent for the field and continue to address the intersectionality among multiple underrepresented groups.^15^

It was also necessary to address the elephant in the virtual room—the participation of men in EWOC! While the 2019 number was not zero, the need for more male allies present was called out by both the founders and attendees. To that end, Stefan Koenig (Genentech) was among the additions to the organizing committee in an effort to send the broader message that male involvement is critical to the success of the EWOC mission and is encouraged and welcomed. As highlighted above, a peer networking session for Allies & Advocates was scheduled to likewise inspire participation. Finally, social media messaging encouraged active inclusion for all genders. From a data standpoint, 16% of registrants for the 2021 EWOC meeting checked the box for using he/him pronouns.^16^ While it is seemingly impossible to identify a target percentage for men at EWOC meetings, we ask that any men reading this article take the message to heart of the value of their attendance and answer the clarion call not just to show up but also to be an active participant at a future EWOC conference. Furthermore, as an organization that aims to be inclusive, we also invite attendees who are nonbinary, recognizing that women are not the only gender minority in organic chemistry and that we are all hoping to learn from and support our nonbinary colleagues.

While the registration fee was low for the in-person version, carrying out the conference in a virtual setting also prompted the question of what is the appropriate registration fee when engaging through a computer screen. Thanks to the aforementioned generosity of our sponsors, we were able to recommend only a small donation to attend, along with the well-received option to register for $0. This approach served to differentiate EWOC from other virtual conferences and render the conference widely accessible. More than 1000 individuals registered to attend #EWOC2021 (“some aspect of virtual is great as many women from around the world can attend without needing to travel. This gives a sense of belonging to the larger community”).^17^ This was true both geographically (essentially all of North America but also five additional continents!) and in the “academic location” of attendees, such as primarily undergraduate institutions (PUIs) where we heard from faculty that they organized watch parties for their undergraduate students (“I’m glad that I could recommend my undergraduate research students attend and that doing so was not cost prohibitive”). Admittedly, this widespread uptake was an unexpected benefit of remote engagement, but having this awareness has had a significant impact on our ongoing conference formats.

### II.B 2022–23: Years Four (Pfizer, Cambridge, MA)^18^ and Five (Amgen, Thousand Oaks, CA),^19^ Hybrid Format

Given our collective learnings from our one in-person conference and two virtual ones, #EWOC2022 meant trying to get the best of both worlds. In so doing, we retained the Thursday all-virtual format that included “Meet Our Sponsors” sessions, workshops, a poster session, and topical networking. For those attending in person, there was a live meet-up near the Pfizer site. Friday was then framed as hybrid, with all speaker presentations and the career panel live-streamed. Additional on-site activities included workshops (which ran concurrently with the career panel), a poster session/cocktail hour, and additional time at breakfast and lunch for networking. Overall, the hybrid platform has been an effective format for maximizing attendance, though certain liabilities remain. In particular, those traveling to the event may have needed to arrive a day early and spend money on a hotel room from which they sat for the Thursday virtual activities. We hosted too many virtual networking sessions, resulting in a bimodal distribution of attendance, leading us to reduce the number of topics for #EWOC2023; similarly, given the number of presenters, the poster sessions were somewhat underserved, frequently leaving the presenters by themselves in their virtual rooms. As a result, while we recognize the value of presenting for those who cannot attend in person, we made the difficult decision to eliminate the virtual poster session in 2023. In general, striking the right balance here will continue to be a challenge for the organizers, and the answer may vary year-on-year. Most participants prefer to attend in person but recognize that might not be feasible for everyone, including themselves. That said, by 2022, many were suffering from Zoom fatigue, which served to reduce the level of online engagement.

It is worth noting that the workshops, in both formats, continue to be a huge selling point for EWOC.^20^ With topics ranging from leading with empathy to how neurodiversity informs inclusive hiring and managing practices, attendees were well-positioned to learn about new areas and achieve career development goals. Gratifyingly, the compendium of workshop presenters came from many different areas: graduate students, faculty, industry sponsors, and professional coaches.

The networking aspect, and associated sense of belonging, was called out repeatedly as a highlight by 2023 EWOC attendees:

- I have been more intentional about how I network and connect with others in the field, as well as how I mentor and request mentorship from others.
- Attending EWOC and networking has helped make me more comfortable talking to strangers who are in similar lines of work.

The impacts extended well beyond the personal level, as feedback around improved communication with Ph.D. advisors, identifying resources for academic courses, and catalyzing a reflexive look at how women feel at a company’s workplace were all called out this year. Perhaps the enduring memory of this most recent conference is the female graduate student in tears thanking Donna for creating EWOC and describing how powerful and impactful this experience was to her.

With the 2022 edition at Pfizer and 2023 at Amgen (including facility tours for interested students and postdocs), in-person EWOC has now been held in three distinct geographies, allowing for rotating local participation. We feel that this is another strength of our EWOC strategy—while individuals may not be able to travel to the conference every year for personal or financial reasons, we hope that, over time, many will be within reasonable traveling distance for a conference. By the same token, this rotation should further diversify the impact of EWOC via a changing group of attendees constantly being added to the network. We have also hosted EWOC in both academic and industrial settings, with each offering its own unique set of benefits. We are excited to announce that #EWOC2024 has been scheduled for June 20–21, 2024, at Merck & Co., Inc., in Rahway, NJ.^21^

### II.C EWOC Chapters

While the establishment of the EWOC conference has been (in our collective opinion) historic, the effort required to sustain such an event effectively limits it to being “only” an annual event. To our delight, there has been an amazing collection of empowered women and allies in organic chemistry who have assumed the torch to ensure that the power of EWOC is felt all year long. This movement has occurred via the formation of eight local chapters across the United States and Canada^22^ (with more on the way) that carry out events ranging from virtual symposia across chapters to mentoring circles to metro area happy hours, all in the name of cultivating a strong sense of belonging across the community of women organic chemists. You can find these chapters on the EWOC Web site and social media platforms to find out more about these events and get involved. Additionally, building on the success of EWOC in North America, the family has been extended to Europe where the first EWOC conference on foreign soil took place at Astra Zeneca in Manchester, England, on June 15, 2023.^23^

## III Founders’ Reflections

While we held our fifth Empowering Women in Organic Chemistry conference in 2023, the journey to get EWOC off the ground and to get here has stretched so much longer. There were the women who came before us who struggled, strived, and thrived in a male-dominated field and have been our role models to persevere. In addition to the blood, sweat, and tears of this group and the additional organizers over the years (including Annaliese Franz, Stefan Koenig, Kimberly Steward, and the local crews at each of our events), we have many outstanding speakers, career panelists, workshop leaders, poster presenters, networking leaders, and attendees to thank for helping turn our minute idea into what we believe is certifiably a global phenomenon, having supported more than 2500 people, that has had and will continue to have a direct impact on addressing the gender distribution in organic chemistry and the field as a whole.

While this perspective has represented our collective spirit, one of the most enduring aspects of empowerment is individualization—the act of suiting the needs of a particular person. As such, we wanted to conclude by sharing about EWOC in our own voices.

**Dr. Ellie Cantor**

My background is in pharmacology, not in chemistry, but I have a passion for supporting and promoting women in science overall. I became aware of EWOC when Donna Huryn, who had been a speaker at one of our programs, reached out to the Philadelphia Chapter of the Association for Women in Science to see if we were interested in participating in the first EWOC conference, as it was to be held in Philadelphia. As a member of the chapter board, I volunteered to be the liaison and thus began my involvement in EWOC. It quickly became apparent to me that the vision of the EWOC team was something critically needed in the chemistry world (not to mention across many other areas of science). I have tremendous respect and admiration for the founders and everyone else who built this remarkable endeavor from concept to reality, and I am awed by how wide a reach we have attained and how quickly the EWOC ethos has spread. I am honored to have been a part of this achievement, and I expect us to continue to have a powerful impact in the future.

**Dr. Margaret Faul**

My journey with EWOC began when Rebecca Ruck reached out to me to write an article on the topic of “Gender Diversity in Process Chemistry” that we published in *Organic Process Research & Development*,^3^ building off a partner article on “Medicinal Chemistry: Where Are All the Women” by Donna Huryn, Wendy Young, and Maria Bolognesi published in *ACS Medicinal Chemistry Letters*.^2^ With the overwhelming response to our article, Rebecca and I knew we needed to do more, and the timing seemed almost perfect since we received the reach out from Donna and Lara on EWOC; with the engagement of Ellie and Mary, the “founders’ team” for the conference was formed! As I look back over the past five years, the opportunity to participate in and advance the vision and mission of EWOC has been one of the highlights of my career. Personally, I have grown through the experiences of working with such amazing colleagues, but I have been humbled by the impact of EWOC on women in the field of organic chemistry. It has provided a unique opportunity for female scientists to share their experiences and for others to learn from those experiences and realize that they have access and support from an “instant network” of colleagues who have worked through similar experiences in building their careers. It has built a community that has allowed the topic of gender diversity in organic chemistry to be openly discussed with valuable insights and opportunities from many in our field of how we can do things differently and why this diversity of opinion is so valuable. So, to everyone who has supported EWOC during our five-year journey, I say thank you, you have made this possible! Organic chemistry is a very rewarding career, and it is important that we continue to build this community for the future.

**Prof. Donna Huryn**

After a career of more than 30 years in chemistry, in both big pharma and academia, I assumed I had experienced all the possible highs (and lows). Little did I know that in 2019 I would look out from the lectern at a scientific meeting and the vision of the audience filled with so many women chemists would be such a new and welcome experience for me that it would bring tears to my eyes. I also realized then, and at every subsequent EWOC meeting, that even when I am “the only” or one of the very few women in the room, I do not need to feel isolated. Through EWOC, we have a community to share, learn from, and rely on, and that idea alone is incredibly empowering, even to those of us who have been in this field for decades. Working with the other Founders, whose dedication, energy, and vision are inspiring, together with our other organizers and supporters, building EWOC is without a doubt a highlight of my career. EWOC serves to remind us that, courtesy of a 2019 tweet from Professor Dani Arias-Rotondo after our first EWOC meeting, “Not only are there a lot of kick-ass women in chemistry, but they are more than happy to help you succeed.”

**Dr. Lara Kallander**

Our vision, a community of women in organic chemistry who support each other, is the experience of being at EWOC. This is also reflected in our logo: five women in a circle holding hands. The design of the conference was very intentional, providing a diverse, inclusive, and innovative environment for attendees to feel a true sense of belonging. The larger purpose behind this is to reduce the leaky pipeline of women leaving organic chemistry. I’m passionate about opening doors for underrepresented voices in science because it is good for scientists and good for innovation in science. We can change the future of organic chemistry, though it takes dedicated effort and opportunities like EWOC to be successful. As a five-year reflection, I’m so grateful to Donna for her bravery, Ellie for her judgment, Rebecca for her social intelligence, Margaret for cultivating hope, and Mary for her fairness, in addition to the many other volunteers who amplified this vision and continue to deliver year after year. I’m awed by the generosity of sponsors to support women in the field of organic chemistry. The EWOC conference is truly a dream come true for me.

**Dr. Rebecca T. Ruck**

When Lara Kallander and Donna Huryn reached out to me about helping to translate their initial EWOC thoughts into reality, I was (1) flattered that they thought I could make meaningful contributions to this effort, (2) energized by the mission in front of us, and (3) downright scared—and correspondingly, motivated—by what the implications would be if it failed. Today, I would describe my state of mind as fulfilled. We write our legacies every day. Historically, to an organic chemist, these legacies would be predicated on achievements such as discovering what would become name reactions and receiving vaunted awards, mile markers for how one has influenced the field. I have had the privilege of being involved in myriad outstanding chemical inventions at my company, some of which have been deployed in processes for preparing life-saving medicines. Nonetheless, I would be very happy if my legacy in the field is that of an innovator in how to render organic chemistry more diverse, equitable, and inclusive, with the creation of EWOC a core component there. I am very proud of what our team of Founders has accomplished and fortunate to share the stage with these five other empowered women.

**Prof. Mary Watson**

As I reflect on five years of EWOC, I’m filled with gratitude and humility. It is amazing to be a part of the organizing team, getting to work with and learn from Donna Huryn, Lara Kallander, Ellie Cantor, Becky Ruck, Margaret Faul, and the other organizers over the years. I am also incredibly thankful for the energy and engagement of the attendees; the atmosphere of EWOC is unlike that of any other conference I’ve attended, and it is due in large part to the mindsets that folks bring. I’m also thankful for and humbled by what EWOC has taught me. As an academic, my world revolves around the experiences of my students, and I was focused on the lack of representation of women in academia. I was convinced that EWOC would be a grad-student centric conference, but so many of the attendees are in industry. It has been a wake-up call for me that women across the career spectrum are looking for an increased sense of community and belonging. I’ve also learned by making mistakes as an organizer of EWOC, and I (and the conference) have received crucial feedback on how we can do better. I am grateful for the folks who were willing to have those hard conversations with us, especially as we got started, so that EWOC could become a more inclusive and supportive event. Finally, personally, EWOC has taught me that it is time to grow up and to stop playing the “little sister” role in rooms full of men, and it is given me exceptional and diverse role models as I develop my own leadership style.

## Notes

This Editorial has been co-published in *The Journal of Organic Chemistry*, *Organic Letters*, *Organic Process Research & Development*, *Organometallics*, *ACS Organic & Inorganic Au*, *ACS Catalysis*, *Biochemistry*, *Journal of Medicinal Chemistry*, *ACS Medicinal Chemistry Letters*, and *ACS Central Science*.
